# From Silver Stains to Deep Learning: Neurohistological Analysis and Digital Mapping of the Brain of Domestic and Laboratory Animals in Veterinary Neuroscience

**DOI:** 10.3390/ani16213486

**Published:** 2026-11-04

**Authors:** Michael Patrignani, Jean-Marie Graïc, Tommaso Gerussi, Valentina Vadori, Livio Finos, Enrico Grisan, Antonella Peruffo

**Affiliations:** 1 Department of Comparative Biomedicine and Food Science, University of Padua, 35020 Legnaro, Italy; michael.patrignani@studenti.unipd.it (M.P.); jeanmarie.graic@unipd.it (J.-M.G.); 2 Department of Agricultural, Food and Forest Sciences (SAAF), University of Palermo, 90128 Palermo, Italy; tommaso.gerussi@unipa.it; 3 Department of Computer Science & Informatics, London South Bank University, London SE1 0AA, UKenrico.grisan@lsbu.ac.uk (E.G.); 4 Department of Statistical Sciences, University of Padua, 35121 Padua, Italy; livio.finos@unipd.it

**Keywords:** neurohistology, comparative neuroanatomy, veterinary neuroscience, domestic animals, cytoarchitecture, stereology, digital pathology, deep learning, brain atlas, multimodal brain mapping

## Abstract

Neurohistological analysis has evolved from descriptive microscopy and classical staining toward quantitative, standardized, and increasingly automated approaches. This narrative review traces this evolution in domestic animals, such as dogs, cats, horses, cattle, pigs, sheep, and goats, with humans and common laboratory animals as methodological comparators, based on the literature retrieved from PubMed, Scopus, and Web of Science. Gross dissection, selective staining, immunohistochemistry, and neuronal tracing progressively expanded the structural and neurochemical information obtainable from the brain, while macroscopic and microscopic morphometry, including design-based stereology, enabled unbiased estimation of brain volumes and cell numbers. Digital microscopy made histological slides computable, and machine learning and deep learning now allow automated segmentation directly from raw images, while magnetic resonance imaging and spatial transcriptomics link histology to connectivity and gene expression. However, direct evidence in domestic animals remains restricted to a few species and brain regions: annotated collections are scarce, data are poorly integrated, and most tools are calibrated on human, mouse, or rat brains. Open resources such as the CytoDArk0 dataset and the CISCA segmentation method are early steps forward. Progress will require species-specific brain atlases, shared protocols, and validated, interpretable methods that treat anatomical differences between species as biological information rather than noise.

## Simple Summary

For over a century, scientists have studied the brain by cutting it into thin slices, staining them, and examining them under the microscope. These methods were first developed and refined mainly on human, mouse, and rat brains. Today, computers and artificial intelligence can analyze thousands of microscope images automatically, measure brain cells with greater precision, and combine this information with brain scans and genetic data into detailed digital maps. However, the brains of domestic animals, such as dogs, cats, horses, cattle, pigs, and sheep, differ markedly in size, shape, and internal organization, and tools designed for mice or humans often perform poorly on them. In this review, we trace how brain tissue analysis has evolved, from early staining techniques to modern computer-based methods, and identify what is still missing for domestic animals: shared image collections, dedicated brain atlases, and methods able to handle their diversity.

## 1. Introduction

Histological analysis has been central to neuroanatomy since the first microscopic observations of neural tissue by Hooke, Malpighi, and van Leeuwenhoek [1,2,3]. In the nineteenth century, selective staining methods, above all Golgi’s *reazione nera* as used by Ramón y Cajal, led to the formulation of the neuron doctrine [4,5,6].

Since then, the histological analysis of the brain has moved from descriptive microscopy to quantitative and increasingly automated analysis. Stereology made it possible to estimate three-dimensional parameters without bias from two-dimensional brain sections [7,8]. Digital microscopy turned brain sections into computable images [9], and machine learning and deep learning now extract relevant features directly from these images [10,11]. More recently, three-dimensional imaging and spatial transcriptomics have begun to combine histology with magnetic resonance imaging, synchrotron tomography, and gene expression data in multimodal brain maps [12,13].

However, these advances have been developed and validated almost exclusively on human, mouse, and rat brains [14,15,16].

Although mammalian brains share a common basic organization, humans, laboratory mice and rats, and domestic animals differ markedly in brain size, cortical architecture, regional proportions, and functional specialization [17,18]. The human brain has a large, highly folded neocortex with extensive association areas. Mice and rats have relatively small, largely lissencephalic brains, with prominent olfactory structures and specialized sensory regions, including the whisker-related barrel cortex in the primary somatosensory cortex [19]. Domestic mammals exhibit substantial variation in brain size, cortical folding, and sensory specialization. In the primary visual cortex of ungulates, the prominence and definition of layer IV vary across species and may differ from the more clearly delineated granular layer found in primates [18]. The organization and functional contribution of corticospinal pathways also differ among mammalian species, reflecting interspecific variation in descending motor control [20].

Consequently, findings obtained in humans, mice, or rats cannot be applied directly to veterinary species. Differences in brain size, cellular organization, and regional anatomy can affect the interpretation of histological findings, the use of immunohistochemical markers, and the performance of image-analysis algorithms trained on human, mouse, or rat data. Likewise, anatomical landmarks and registration procedures developed for one species may not identify the corresponding structures in another.

In addition, brain tissue from many veterinary species is scarce and of heterogeneous origin [21], and standardized brain atlases, annotated datasets, and analytical pipelines are lacking [22]. Methods developed in humans, mice, and rats therefore need to be adapted and validated before they can be used in veterinary neuroscience. At the same time, the anatomical diversity of these brains offers a natural test of whether methods developed in a few reference species apply to mammals in general [23].

These gaps are also clinically relevant, because many common neurological diseases of domestic animals are diagnosed and characterized on the basis of brain lesions [24,25]. In dogs and cats, these include meningoencephalitis of unknown origin, intracranial tumors, cerebrovascular disease, and age-related changes such as canine cognitive dysfunction [26,27,28,29,30]. In other domestic animals, infectious, metabolic, nutritional, and toxic encephalopathies also require clinical signs to be interpreted together with neuroanatomical and neuropathological findings [31,32]. In all these conditions, histology and immunohistochemistry of the brain remain essential to characterize lesions and to interpret MRI findings (Section 7). Comparative brain mapping is clinically useful for three reasons. First, species-specific brain atlases are needed to localize lesions on MRI and histological sections and to distinguish them from normal variation, such as age-related brain atrophy, which differs among dogs with different skull conformations (Section 7). Second, comparison with human, mouse, and rat brains shows which diagnostic markers, imaging criteria, and analytical tools can be transferred to domestic animals and which require species-specific validation. Third, several spontaneous brain diseases of domestic animals, such as canine gliomas and canine cognitive dysfunction, resemble human disorders, so comparative brain mapping can support both veterinary diagnosis and translational research within a One Health perspective [27,30].

Several reviews have described the evolution of methods for studying the human brain, including neuromorphometry [33], tract tracing [34], architectonic mapping from Brodmann to modern multimodal approaches [35,36], human brain atlases [37], and deep learning in medical image analysis [11,38]. These reviews, however, focus on the human brain or on single methods, and, to our knowledge, none has examined the evolution of neurohistological analysis of the brain, from classical to computational methods, from the perspective of species of veterinary interest. We focus on the brain because most of the methods discussed here, such as cytoarchitectonic analysis, stereotaxic and digital atlases, and multimodal brain mapping, were developed for the brain, and because differences between species, particularly in cortical organization and folding, are most marked there. In addition, many common neurological diseases of domestic animals that require histological characterization mainly affect the brain. The spinal cord and peripheral nervous system, although clinically important, have distinct anatomical features, methods, and diagnostic problems and deserve separate treatment.

The aims of this review are: (i) to describe how neurohistological methods for studying the brain have evolved, from classical staining to quantitative, computational, and multimodal approaches, and how they have been applied to species of veterinary interest; (ii) to identify the main factors that limit the use of methods developed in humans, mice, and rats in these species; and (iii) to outline the resources needed for future research, such as species-specific brain atlases, shared datasets, and validated analytical methods. We also emphasize that anatomical differences between species reflect real biological variation and should be preserved and studied, rather than removed during data analysis.

## 2. Methods

### 2.1. Research Question

This narrative review addresses the following research question: How have neurohistological methods for studying the brain evolved in species of veterinary interest, and what are the current challenges and opportunities in applying quantitative, machine learning, deep learning, and multimodal approaches to the brains of species of veterinary interest?

### 2.2. Definitions and Scope

In this review, the term computational brain mapping refers to the creation of digital maps of the brain of an individual animal or of a species. These maps are built from anatomical data, such as histological sections, magnetic resonance images, or molecular data, and each brain structure is assigned a defined position within a common three-dimensional coordinate system. This allows brain structures to be measured quantitatively and compared across individuals and species [16,37,39].

For the purposes of this review, species of veterinary interest are the domestic animals for which brain studies were retrieved, namely the dog (*Canis lupus familiaris*), cat (*Felis catus*), horse (*Equus caballus*), cattle (*Bos taurus*), pig (*Sus scrofa domesticus*), sheep (*Ovis aries*), goat (*Capra hircus*), and llama (*Lama glama*). Studies on humans, non-human primates (*Macaca mulatta*, *Pan troglodytes*), and common laboratory animals, namely mice (*Mus musculus*), rats (*Rattus norvegicus*), Syrian hamsters (*Mesocricetus auratus*), and rabbits (*Oryctolagus cuniculus*), were included only when they introduced a brain research method later applied to domestic animals or served as a direct comparator [40]. Aquatic, exotic, and wild species were not included. The species included in each study are listed in Tables S1 and S2. The review was restricted to the brain; studies focusing on the spinal cord or peripheral nervous system were not included.

### 2.3. Literature Search Strategy

The literature search was conducted in PubMed/MEDLINE, Scopus, and Web of Science Core Collection. Google Scholar was used additionally, in a targeted and non-systematic way, to identify historical works, monographs, and book chapters not indexed in bibliographic databases; because these searches were not systematic, the number of Google Scholar results was not recorded. Database searches covered publications from January 1990 to June 2026, and the last search was performed on 15 June 2026. The search strategy combined three groups of terms with the Boolean operator AND: (i) terms related to the brain and to neurohistological and morphometric analysis (e.g., brain, cerebral cortex, cytoarchitecture, Nissl, Golgi, immunohistochemistry, stereology, morphometry); (ii) terms related to digital and computational methods and brain mapping (e.g., digital histology, whole-slide imaging, image analysis, machine learning, deep learning, segmentation, brain atlas, magnetic resonance imaging); and (iii) terms related to species of veterinary interest (e.g., dog, cat, horse, cattle, sheep, goat, pig). Within each group, synonyms were combined with the operator OR. The complete search strings used for each database are reported in Table S3. Peer-reviewed articles, books, and book chapters were eligible if they were published in English or had an English abstract (with the exception of foundational historical works; see below), and if they concerned the brain and addressed one of the following: neurohistological techniques applied to brain tissue; stereology or morphometry of the brain; computer-assisted or digital histology of brain sections; machine learning or deep learning applied to histological or neuroimaging data of the brain; or brain atlas development or multimodal brain mapping.

In each case, the work had to concern the brain of species of veterinary interest within a comparative framework, or describe a brain research method subsequently applied to these species. Primary research articles, reviews, and methodological papers were all considered. As the only exception to the language criterion, and given the historical scope of the review, foundational contributions (e.g., original descriptions of neurohistological staining methods) were included regardless of publication date and language (Section 2.4).

Studies were excluded if they concerned exclusively humans or laboratory mice and rats and lacked methodological or comparative relevance to the brains of species of veterinary interest. Conference abstracts and the grey literature were also excluded, with the exception of publicly accessible online brain repositories, namely the Comparative Mammalian Brain Collection (brainmuseum.org), BrainMaps.org, the Scalable Brain Atlas, the Digital Brain Bank, and braincatalogue.org, which were consulted directly and are described in Section 6 and Section 8.2.

### 2.4. Study Selection

Three authors (A.P., J.-M.G., and T.G.) independently screened the records retrieved from the database searches against the eligibility criteria described in Section 2.3, first by title and abstract and, when necessary, by full text. Disagreements were resolved by discussion, and the final selection was agreed upon by consensus among the three authors. Backward citation tracking was performed by screening the reference lists of the included articles and of previous reviews; forward citation tracking was not performed systematically. As this is a narrative rather than a systematic review, the number of records retrieved, screened, and excluded at each stage of the selection process was not systematically recorded.

Because abstract and keyword indexing in bibliographic databases is incomplete for publications prior to 1990, historical sources could not be reliably retrieved through database searches. Foundational works published before this date were therefore identified through Google Scholar, reference monographs, and the reference lists of the included studies, and were included when they introduced a technique, concept, or reference atlas subsequently adopted in brain research (e.g., the original descriptions of the Golgi, Nissl, and myelin staining methods), irrespective of language or study design.

### 2.5. Limitations of the Review

This review has several limitations.

First, its broad temporal and taxonomic scope required selective coverage; relevant contributions published in languages other than English, or available only as the grey literature, may not have been fully captured despite the search and selection strategy described in Section 2.3 and Section 2.4.

Second, the heterogeneity of species, study designs, and brain research methods precluded formal quantitative synthesis, so the integration of evidence necessarily involves an element of interpretive judgment.

Third, given the rapid pace of development in machine learning and computational neuroanatomy, some very recent contributions may not be represented.

Fourth, the scarcity of openly accessible brain datasets from species of veterinary interest (Section 8.2) prevented systematic benchmarking of methods across species, and the resulting methodological comparisons are therefore necessarily qualitative.

Fifth, as this is a narrative review, the number of records retrieved and screened at each stage of the selection process was not systematically recorded (Section 2.4), which limits the reproducibility of the selection.

Finally, annotation biases and the anthropocentric assumptions embedded in many existing analytical frameworks may influence the interpretation of brain data from species of veterinary interest in ways that are difficult to fully account for within the current evidence base; addressing this issue in depth lies beyond the scope of the present review, whose aim is to describe the current status of brain research in species of veterinary interest.

## 3. Evolution of Neurohistological Techniques: From Classical Staining to Three-Dimensional Imaging

An early precursor of systematic comparative neuroanatomy was Félix Vicq d’Azyr, whose standardized sectioning, species comparisons, and iconography of cerebral structures anticipated principles of quantitative neuroanatomy [41]. Selective staining techniques then made it possible to visualize specific components of neural tissue (Table 1 and Table 2). Golgi’s *reazione nera*, which impregnates a small, apparently random subset of neurons in their entirety (as shown in the barrel cortex of the mouse, *Mus musculus*) [42], provided unprecedented insight into neuronal morphology and contributed to the neuron doctrine formulated by Ramón y Cajal on the nervous system of humans and other vertebrates [4,5,6,43].

Nissl staining, which labels the rough endoplasmic reticulum of neurons and the nuclei of glial and endothelial cells, became the basis for the study of cortical lamination, cell density, and cytoarchitecture, and for comparative cortical parcellation, established by Brodmann in the human brain and in many other mammalian species [44,45].

Myelin stains, developed by Weigert on human brain tissue and later combined with a Nissl counterstain by Klüver and Barrera in the rhesus monkey (*Macaca mulatta*) and humans, and silver methods such as the Bielschowsky impregnation, applied to rat and human brain tissue, revealed fiber systems, white matter architecture, and neurofibrils [46,47,48]. Nissl and myelin preparations remain the primary substrate for automated segmentation, cytoarchitectural analysis, and atlases, including in ungulates (e.g., the horse), non-human primates, and humans [18,49,50].

From the 1940s onward, histochemistry and immunohistochemistry added a neurochemical dimension to the same sections, allowing the identification of enzymes, neurotransmitters, and cell populations that conventional stains could not distinguish [51,52,53,54,55]. Cholinesterase histochemistry, first described in rat tissues, introduced the principle of translating the enzymatic identity of cells into tissue maps, a precursor of marker-based segmentation [52], and immunohistochemistry enabled the mapping of neurotransmitter systems, neuronal subpopulations, and glial phenotypes, initially mostly in the rat brain [56,57,58,59]. In veterinary species, however, antibodies developed for human, mouse, or rat tissues show variable specificity and require careful validation [22] (for a review, see [60]).

Connectivity was first studied with the silver impregnation of degenerating axons developed by Nauta and Gygax in the rat brain [34,61,62,63]. From the 1970s, this approach was replaced by tracers actively transported along axons and injected in vivo, as in the mouse, rat, and cat [34,64,65], and by carbocyanine dyes applicable to fixed post-mortem tissue, as in the rat spinal cord [66]. Tract tracing has been applied to sheep, dogs, cats, and rabbits [67,68,69,70,71,72], but in vivo injections are rarely justifiable in these species, which makes post-mortem methods particularly relevant for material from necropsies. Diffusion-weighted imaging (DWI) and tractography now allow the non-invasive reconstruction of putative white matter pathways, including in ex vivo brains of large animals such as the sheep, although they do not resolve individual axons or synaptic connections [73,74].

At the macroscopic level, white matter tracts can also be studied by anatomical dissection. In fiber dissection (Klingler’s technique [75]), formalin-fixed brains are usually frozen and thawed before the white matter fibers are separated by blunt dissection, revealing the course and relationships of the major tracts in three dimensions. This method has been used to describe the major white matter tracts in the horse, cat, and dog [76] and in the pig [77], and the association fasciculi in cattle, pigs, and rabbits [78]. Although its resolution is limited to macroscopic fiber bundles, dissection provides a direct anatomical reference for the interpretation of tractography.

Because histological analysis is inherently two-dimensional, three-dimensional structures were first reconstructed from serial sections, as in the albino rat, and later with registration approaches applied to rat, cat, and human brain data [79,80,81]. Tissue-clearing methods such as CLARITY [82] and iDISCO [83], developed mainly on the mouse brain [84] and combined with light-sheet or two-photon microscopy, now allow volumetric imaging of entire brain regions or whole small brains [85,86], complementing electron microscopy-based connectomics of the mouse visual cortex [87]. Their application to the large brains of cattle and horses requires substantial adaptation of protocols, but is expected to increase the availability of atlases and datasets for comparative analysis. The main methodological transitions are summarized in Table 1, and the principles, advantages, disadvantages, and species of application of each method in Table 2.

**Table 1:** Summary of the main historical and methodological transitions in neurohistological analysis of the brain relevant to veterinary neuroanatomy. For each phase, the table reports the historical phase or transition, the main methodological innovation, its relevance to the study of the brain in veterinary species, the primary limitations, the section of this review in which it is discussed, and the key references. Transitions are presented in broad chronological sequence, from early optical microscopy to contemporary multimodal computational brain mapping.

Historical Phase/Transition	Main Methodological Innovation	Impact on the Study of the Brain in Veterinary Species	Main Limitations	Section	Key References
1. Early microscopy and classical neurohistology (17th–19th century)	Development of optical microscopy and first histological observations of nervous tissue, including the brain	Enabled the first descriptions of brain microstructure and comparative observations across species	Limited staining specificity; absence of standardized analytical frameworks	Section 1 and Section 3	[1,2,3]
2. Selective staining and neuron doctrine	Golgi staining methods and cytoarchitectural analysis of the brain	Supported systematic investigation of the organization of the cerebral cortex in domesticated and non-model species	Predominantly qualitative interpretation and manual annotation	Section 3	[4,5,88,89]
3. Descriptive comparative neuroanatomy	Application of histology to the brain of veterinary species	First comparative descriptions of bovine, equine, canine, and porcine brains	Poor reproducibility; lack of species-specific atlases; difficult cross-species comparisons	Section 3	[45,90,91]
4. Quantitative stereology and morphometry	Probabilistic sampling, disector principle, optical fractionator	Unbiased estimation of neuronal populations, cortical volumes, and cytoarchitectural parameters of brain regions across species	Labor-intensive workflows; limited applicability to large heterogeneous datasets	Section 4	[7,92,93,94,95,96,97]
5. Digital histology and computational imaging	Digitization of microscopy and image analysis algorithms	Histological sections of the brain became computable datasets enabling automated measurements and morphometry	Dependence on computational infrastructure and annotated datasets	Section 4	[9,98]
6. Machine learning and AI-based neurohistology	Deep learning, automated segmentation, hierarchical feature extraction	Allowed automated cell segmentation and quantitative analysis of histological images of the brain from mammalian species, including species of veterinary interest	Limited transferability of models across phylogenetically diverse brains	Section 4 and Section 5	[11,99,100]
7. Multimodal computational brain mapping	Integration of histology with MRI, tractography, transcriptomics, and connectomics	Cross-species alignment of brain organization and development of comparative digital atlases	Fragmented and insufficiently standardized veterinary datasets	Section 6	[14,15,101,102]

**Table 2:** Main neurohistological, tract-tracing, and imaging methods discussed in this review. For each method, the table summarizes its principle, main advantages and disadvantages, the species in which it has been applied (examples cited in the text), and the key references.

Method	Principle	Advantages	Disadvantages	Species of Application (Examples)	Key References
Golgi impregnation (*reazione nera*, Golgi–Cox)	Silver chromate impregnation of a small, apparently random subset (~2%) of neurons in their entirety	Complete visualization of soma, dendrites, spines, and axon; basis of the neuron doctrine and of quantitative dendritic morphometry	Unpredictable, poorly understood selectivity; capricious results; unsuitable for estimating cell populations	Humans and other vertebrates; mouse (barrel cortex); rabbit (cortical neurons)	[4,5,6,42,43,103]
Nissl staining	Basic aniline dyes binding nucleic acids (rough endoplasmic reticulum, ribosomes) of neuronal somata and glial and endothelial nuclei	Simple, inexpensive, and robust across species and post-mortem material; reveals lamination, cell density, and cytoarchitecture; main substrate for automated segmentation and atlases	No visualization of neuronal processes; distinction of neurons and glia requires expertise or dedicated algorithms; cell clustering hampers automated counts	Humans and a broad range of mammals; horse, chimpanzee, macaque; ungulates; cattle	[18,44,45,49,50,104]
Myelin staining (Weigert, Luxol fast blue, Klüver–Barrera)	Selective staining of myelin sheaths, alone or combined with a Nissl counterstain	Visualization of myelinated fiber systems and white matter architecture; combined cell and fiber staining on the same section	No information on individual axons or on the direction of projections	Human (Weigert); rhesus monkey (Macaca mulatta) and human (Klüver–Barrera)	[46,47,48]
Histochemistry and immunohistochemistry	Enzymatic reaction or antibody–antigen binding visualized by chromogenic or fluorescent labels	Identification of enzymes, neurotransmitters, neuronal subpopulations, and glial phenotypes on the same sections used for morphology	Antibodies developed for human, mouse, or rat tissues show variable specificity in other species; post-mortem delay and fixation affect antigenicity; extensive validation required	Rat (cholinesterase activity, neurotransmitter systems); sheep (orbitofrontal cortex)	[22,51,52,53,54,55,56,57,58,59,60,105]
Degeneration silver impregnation (Nauta–Gygax)	Selective silver impregnation of axons degenerating after an experimental lesion	First experimental approach to long-range anatomical connectivity	Requires experimental lesions in living animals; largely replaced by axonal tracers	Rat (experimental lesions)	[34,61,62,63]
Axonal tract tracing (horseradish peroxidase, fluorescent tracers)	In vivo injection of tracers actively transported along axons and detected post-mortem	Retrograde and anterograde reconstruction of circuits; multiple labeling of collateral projections	Requires in vivo injections, which are technically and ethically difficult or impossible in large, protected, or client-owned animals	Mouse, rat, cat, sheep, dog, rabbit	[34,64,65,67,68,69,70,71,72]
Lipophilic carbocyanine dyes (e.g., DiI)	Passive lateral diffusion of dyes along cell membranes in fixed tissue	Applicable to post-mortem material, including tissue from necropsies	Short tracing distances; very slow diffusion	Human brain; rat spinal cord	[63,66]
Fiber dissection (Klingler’s technique)	Blunt dissection of white matter fibers in formalin-fixed brains, usually after freezing and thawing	Direct three-dimensional visualization of the course and relationships of major white matter tracts; inexpensive; anatomical reference for tractography	Limited to macroscopic fiber bundles; does not resolve individual axons or small tracts; destructive and operator-dependent	Horse, cat, dog, pig, cattle, rabbit	[75,76,77,78]
Diffusion-weighted imaging (DWI) and tractography	MRI-based estimation of water diffusion to infer the orientation of white matter fibers	Non-invasive; whole-brain coverage; applicable in vivo and ex vivo to large brains	Indirect, non-microscopic resolution; requires histological validation	Sheep; other large mammals ex vivo	[73,74,105]
Serial section reconstruction	Three-dimensional reconstruction from aligned consecutive histological sections	Three-dimensional information from standard histological preparations	Labor-intensive; section distortion and registration errors	Albino rat; rat, cat, and human (image registration)	[79,80,81]
Tissue clearing combined with light-sheet or two-photon microscopy (e.g., CLARITY, iDISCO)	Chemical reduction in light scattering while preserving molecular labeling, followed by volumetric optical imaging	Volumetric imaging of entire brain regions or small whole brains without physical sectioning	Protocols optimized for the mouse brain; large brains (cattle, horse) require adaptation of clearing times, reagents, and imaging parameters	Mouse	[82,83,84,85,86]
Electron microscopy-based connectomics	Dense reconstruction of neurons and synapses from serial electron microscopy images	Synaptic-resolution wiring diagrams of cortical volumes	Restricted to small tissue volumes; very large datasets and computational costs	Mouse (visual cortex)	[81,87]
Design-based stereology (Cavalieri principle, disector, optical fractionator)	Systematic random sampling of sections for unbiased estimation of three-dimensional parameters	Unbiased estimates of cell number, volume, and density, independent of cell shape and size	Time-consuming and operator-dependent; difficult to apply to large brains; sensitive to tissue shrinkage and fixation	Human and rat hippocampus; human cerebral cortex; sheep, goat, and rabbit (brain volumes)	[7,8,33,92,93,94,95,96,97,106,107,108,109,110]

## 4. From Quantitative Morphometry to Computational Neuroanatomy

### 4.1. Macroscopic Morphometry of the Brain

Macroscopic morphometry measures the size and shape of the whole brain and of its main subdivisions, such as the cerebral hemispheres, cerebellum, grey and white matter, and ventricles, on gross specimens, serial slices, or magnetic resonance images. Volumes can be estimated without bias with the Cavalieri principle, which combines systematic sampling of slices with point counting on their cut surfaces [7,97]. This approach has been applied, for example, to the brain of the sheep [108], to the cerebral hemispheres, grey and white matter, and lateral ventricles of sheep and goats [109], and to the brain, cerebral hemispheres, and cerebellum of New Zealand rabbits [110]. Combined with magnetic resonance imaging, it has also been used to describe age-dependent changes in albino rats [111].

### 4.2. Microscopic (Histological) Morphometry and Stereology

At the microscopic level, design-based stereology provides unbiased estimates of cell number, density, and volume through systematic random sampling [8,92,95,106]. The optical fractionator and the disector were first applied to the human and rat hippocampus [8,96,97,107]. Haug [33] showed that apparent age-related neuronal loss in the human cerebral cortex often reflected tissue shrinkage due to fixation, underlining the need to account for tissue processing when comparing quantitative data. Because stereology is labour-intensive, particularly in large brains (Table 2), computational imaging methods were progressively adopted.

### 4.3. Digital Image Analysis and Machine Learning

The digitization of microscopy was gradual, from the first computer-assisted microscopes and early digital morphology [103,112,113] to scanning microscopes [114], CCD cameras [115,116], and machine vision [117,118], and was followed by semi-automated measurement, morphometry, and statistical analysis [9,98]. The computer-microscope of Glaser and Van der Loos [103], which reduced the reconstruction of Golgi-stained rabbit cortical neurons from hours to minutes, marked the passage from analog observation to computer-based measurement. Early algorithmic morphometry relied on handcrafted descriptors that increased objectivity but transferred poorly across staining protocols and species [119,120], a limitation particularly evident in comparative neuroanatomy [121,122,123,124].

Machine learning and deep learning, applied in supervised, unsupervised, self-supervised, and weakly supervised settings [125], shifted image analysis from rule-based quantification to learning features directly from images. Convolutional neural networks, U-Net-based segmentation models, and transformer architectures are now the main tools for the segmentation and classification of histological images [10,11,99,126,127,128,129]; their contributions and limitations, including the limited biological interpretability of learned features [130,131,132], are summarized in Table 3, and strategies for veterinary datasets are discussed in Section 8.

## 5. Computational Histology and Cytoarchitectural Analysis in Veterinary Neuroanatomy

Early computer-assisted histology focused on the quantification of Nissl-stained sections: the Grey Level Index, introduced by Schleicher, Zilles, and Wree in the rat brain, measures the proportion of each sampling field occupied by Nissl-stained cell bodies; it reflects cell packing density and supported the detection of cortical boundaries, but it depends on staining and image acquisition and is not an unbiased cell count [143]. This approach was complemented by receptor architectonics, which added a neurochemical dimension to cortical parcellation in the human and rat brain [35,36,144].

Similar approaches have been used to estimate neuronal and neuropil parameters across cortical areas in humans, great apes, and Old World monkeys [145,146], but rarely in large-brained species such as the horse and artiodactyls [50,147]. In parallel, cell segmentation algorithms were developed for confocal images of human tissues and *Caenorhabditis elegans* embryos [148] and for brightfield images of mouse brain sections [149], including methods for resolving clustered neurons in Nissl-stained sections of the bovine cerebellum [104].

Machine learning and deep learning methods were then developed specifically for cell recognition and segmentation in human pathology [150,151,152] and in neuroanatomy, for example on macaque cortical sections and cultured mouse and rat neurons [153,154], shifting histological analysis toward high-throughput automated processes in human histopathology [155]; this approach has also been extended to disease classification from human neuroimaging data [156,157]. In veterinary histopathology, deep learning is also increasingly applied beyond the nervous system and across species, for example to the automated diagnosis of seven canine skin tumor types on hematoxylin and eosin-stained whole-slide images [158], the histological classification of canine and feline lymphoma [159], and the classification of canine testicular tumors [160].

These developments are often discussed within the frameworks of computational pathology [161] and computational neuroanatomy [162,163], and overlap with computational neuroscience, the broader field of mathematical analysis and modelling of neural systems [164]. In this review, we use the term computational histology for the application of digital image analysis, machine learning, and deep learning to digitized histological data. In veterinary neuroscience, the open CISCA framework for cell segmentation in whole-slide images and the CytoDArk0 dataset of annotated Nissl-stained brain sections from several mammalian species, including mouse, chimpanzee, macaque, and cattle (*Bos taurus*), represent early steps toward addressing annotation scarcity [100].

Applied in veterinary neuroanatomy, these methods have revealed patterns of cortical organization related to motor adaptation, evolutionary divergence, and functional specialization. In the horse, the laminar organization of the motor cortex differs markedly from that of primates, limiting the transfer of canonical neocortical models to distant lineages [50], while in the sheep the integration of cytoarchitectural, neurochemical, and DWI data improved the interpretation of species-specific features [73,105]. Comparisons across ungulates and primates, and across domestic dog lineages, have revealed structural differences potentially related to evolutionary history, ecological adaptation, or functional specialization [18,165].

Statistical modeling has further extended the analysis of digitized tissue, for example to sexual dimorphism in the bovine cerebellum [166], hemispheric asymmetry in the area 44 homolog of the chimpanzee (*Pan troglodytes*) [167], the effect of estrogens on the aging brain of the dog [168], and the motor cortex of the domestic pig and the wild boar [169] (see also Table 4).

**Figure 1:**
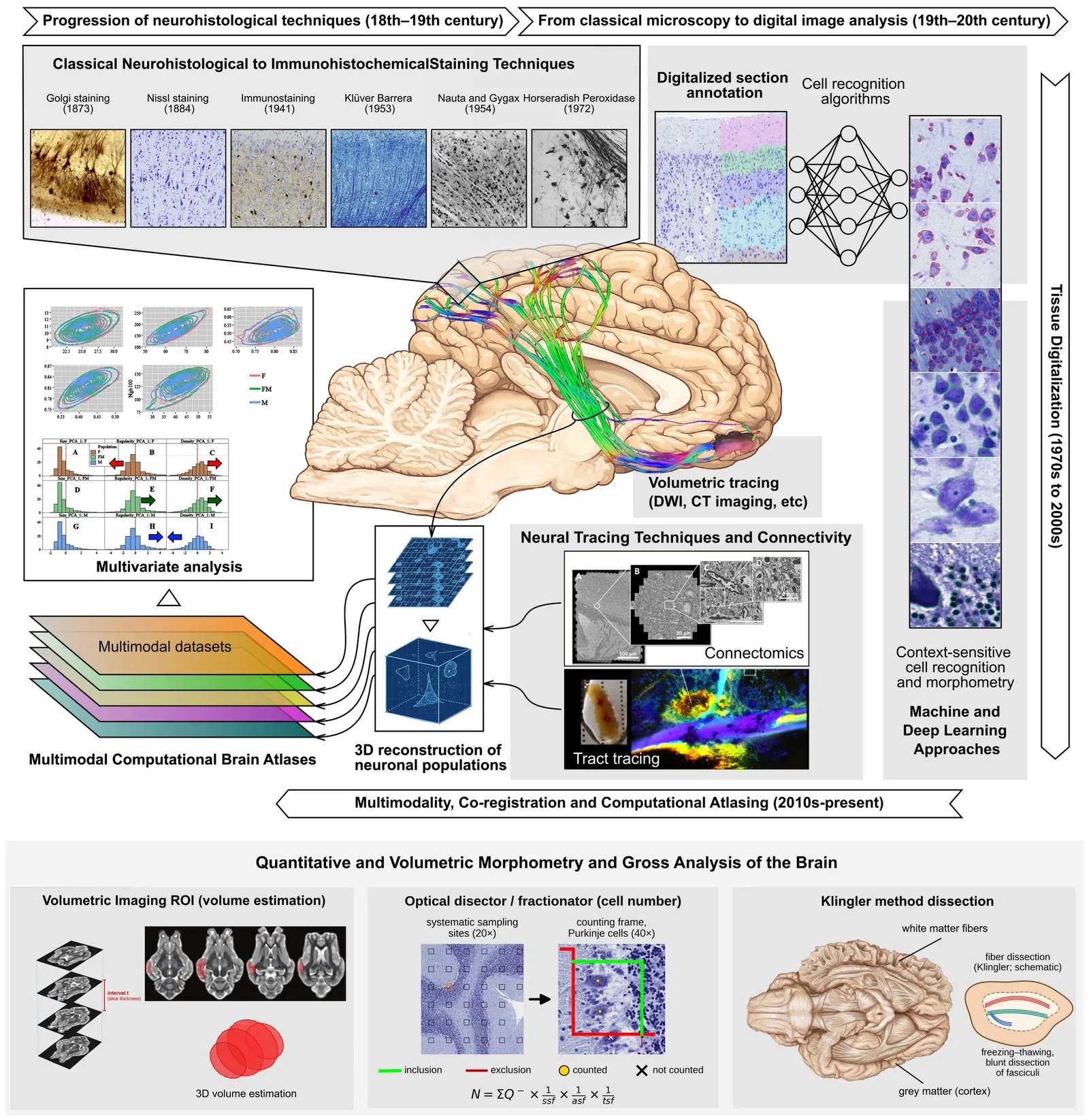
From classical neurohistology to deep learning: methodological evolution in neurohistological analysis and computational brain mapping. The figure summarizes the progressive transition from classical staining and tracing techniques toward quantitative image analysis, automated cell recognition, three-dimensional reconstruction, and multimodal computational analysis. The surrounding banners indicate four broad, partly overlapping methodological phases: progression of neurohistological techniques (18th–19th century), transition from classical microscopy to digital image analysis (19th–20th century), tissue digitization (1970s–2000s), and multimodality, co-registration, and computational atlasing (2010s–present). Upper left: classical neurohistological, histochemical, and immunohistochemical techniques, shown with their year of introduction: Golgi staining and Nissl staining for the morphological characterization of neurons, immunostaining for molecular and neurochemical information, Klüver–Barrera staining of myelinated fibers and cells, Nauta–Gygax silver impregnation of degenerating axons, and horseradish peroxidase tract tracing. Upper right: annotation of digitized sections and cell recognition algorithms. Right column: context-sensitive cell recognition and morphometry through machine and deep learning approaches. Middle left: multivariate analysis for the quantitative extraction and comparison of morphological features across neuronal populations and brain regions. Centre: volumetric imaging of the brain, including diffusion-weighted imaging (DWI) tractography of white matter pathways and computed tomography (CT). Lower right: neural tracing techniques, including connectomics and tract tracing, supporting connectivity mapping. Lower centre: three-dimensional reconstruction of neuronal populations from serial sections. Lower left: integration of heterogeneous datasets into multimodal computational brain atlases. Bottom panel: quantitative and volumetric morphometry and gross analysis of the brain. Left: volume estimation of a region of interest (ROI, red) on serial magnetic resonance images of the sheep (*Ovis aries*) brain, acquired at a constant slice interval t, according to the Cavalieri principle (V = t × ΣA, where ΣA is the sum of the ROI areas on the sampled slices), with a schematic three-dimensional reconstruction of the estimated volume. Centre: the optical fractionator, illustrated on Nissl-stained sections of the chimpanzee (*Pan troglodytes*) cerebellum, with systematic sampling sites (original magnification 20×) and an unbiased counting frame with inclusion (green) and exclusion (red) lines applied to Purkinje cells (40×), shown for illustration (N = ΣQ^−^ × 1/ssf × 1/asf × 1/tsf, where ΣQ^−^ is the number of counted cells and ssf, asf, and tsf are the section, area, and thickness sampling fractions). Right: original schematic illustration of fiber dissection according to Klingler’s method in the bovine (*Bos taurus*) brain, in which white matter fasciculi are exposed by blunt dissection after freezing and thawing of the fixed brain. All images in the bottom panel are original. Together, these approaches illustrate the methodological continuum from descriptive neurohistology to quantitative, high-throughput, and machine-learning-assisted analysis in veterinary neuroscience.

Together, these studies show that digital histology supports both automated cell detection and quantitative statistical analysis in developmental, evolutionary, comparative, and pathological contexts, providing a foundation for addressing the challenges discussed below. The methodological evolution from classical neurohistology to computational analysis is summarized in Figure 1.

## 6. From Histological References to Computational Multimodal Brain Atlases

Brain atlases have evolved from static reference maps to digital systems that integrate several imaging modalities and biological data. Classical atlases were based on histological sections, gross dissection, documentation of gyri and sulci, and expert cytoarchitectural parcellation, as in the cytoarchitectural map of the sheep cortex and the stereotaxic atlas of the beagle brain [170,171]. These atlases organized information mainly around morphological and cytoarchitectural reference spaces and could integrate different datasets less easily than modern digital platforms [37].

Modern brain maps combine several spatial scales and data types, including histology, MRI, connectivity, gene expression, and cellular data, within a common three-dimensional reference space, as in the Allen Mouse Brain Common Coordinate Framework [16].

This integration can reveal relationships between cytoarchitecture, molecular organization, and gene expression that no single method can show, as demonstrated in the human brain [172].

A key step was the Allen Mouse Brain Atlas (*Mus musculus*), which mapped genome-wide gene expression onto a standard anatomical reference [14,16].

The Allen Human Brain Transcriptome Atlas extended this approach to the human brain [173], and the Human Connectome Project provided a multimodal framework for mapping human brain structure, function, and connectivity [15,101].

In small brains, automated whole-brain histological imaging and quantification are now possible, as shown by high-throughput 3D histopathology pipelines in mice [174]. Surface-based cortical atlases have also been built for non-human primates, such as the rhesus monkey (*Macaca mulatta*) [102].

These surface-based atlases provide standard spatial frameworks for comparing cortical areas and neuroimaging data across individuals and species.

In other species, atlases often rely on a single method; for example, the Catlas is an MRI-based three-dimensional cortical atlas of the domestic cat [175].

Spatial transcriptomics, connectomics, and high-throughput imaging have extended brain atlases beyond morphology, mainly in mouse and human tissues [176,177,178], and describe the brain as a multiscale system in which cell identity, gene expression, connectivity, and topology interact, as shown by connectomic studies in the mouse and by the reconstruction of rat neocortical microcircuits [179,180,181].

Although deep learning has shown potential for disease classification and data integration in human neuroimaging [157,182,183,184], comparable resources are still limited in veterinary neuroscience. Species-specific resources do exist, but their scope and methods vary widely. Examples include stereotaxic and neuroimaging atlases of the dog [171,185], MRI-based anatomical references for the horse [186,187], an MRI-based atlas of the pig brain [188], and ongoing work toward standard sheep brain atlases [189]. MRI-based descriptions of normal brain anatomy are also available for the cat [190] and, among laboratory species, for the rabbit at 3 T [191].

**Figure 2:**
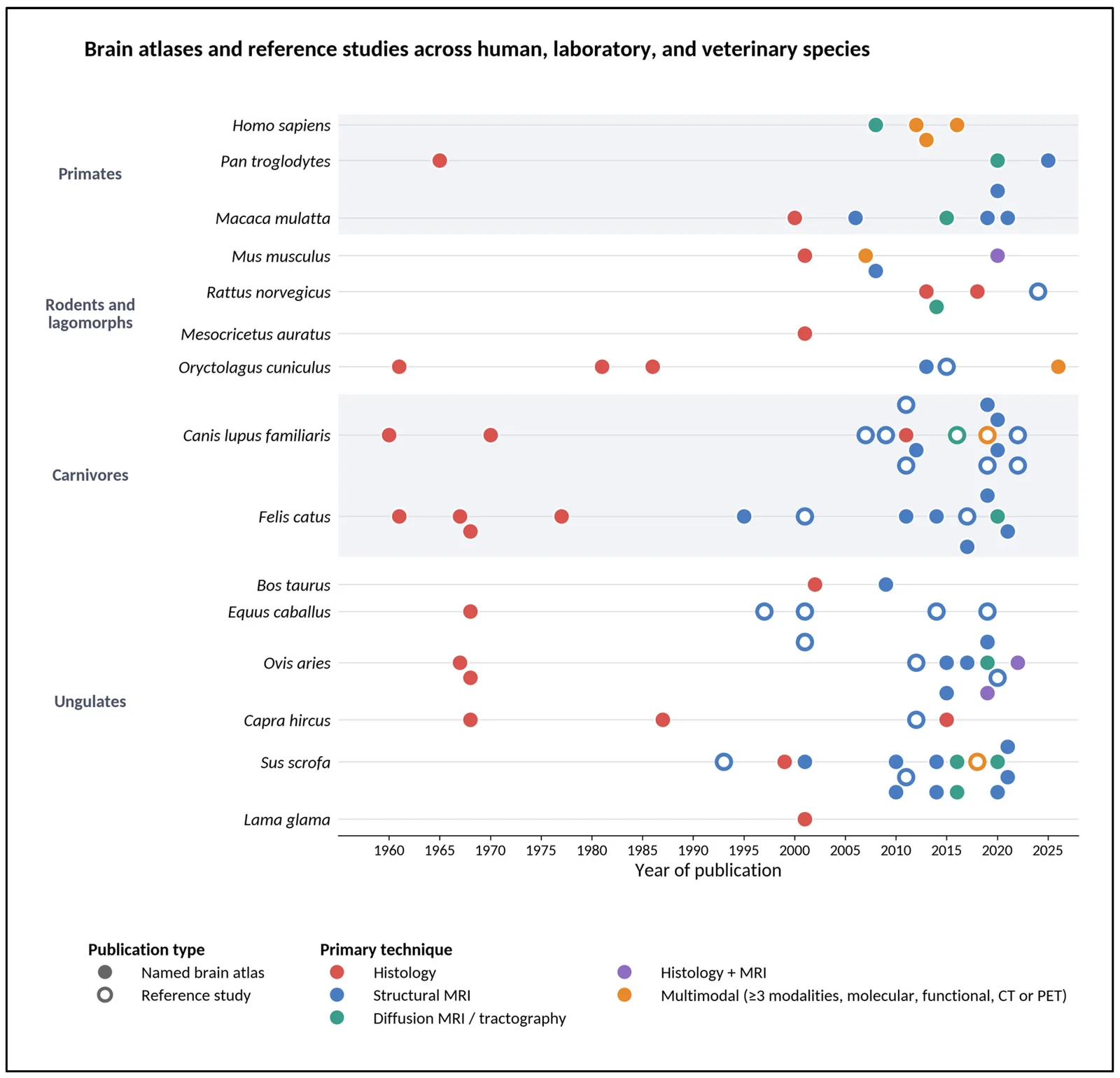
Brain atlases and reference studies across human, laboratory, and veterinary species (1960–2026). Each symbol corresponds to one publication for one species, positioned by publication year (*x*-axis) and species (*y*-axis); publications covering several species are shown once for each species, and symbols for publications from the same or adjacent years are stacked vertically to avoid overlap. Species are grouped by taxonomic category. Filled circles indicate named brain atlases (whole-brain or, in some cases, region-specific); open circles indicate other studies providing reference-level anatomical data on the brain of that species. The same inclusion criteria were applied to all species, including those for which a complete atlas is available. Colors indicate the primary technique: histology, structural MRI, diffusion MRI or tractography, histology combined with MRI, or multimodal approaches (three or more modalities, or including molecular, functional, CT, or PET data). The figure illustrates general trends and is not an exhaustive survey of the literature; full references for each data point are listed in Table S1.

A few multispecies repositories are available—the Comparative Mammalian Brain Collection (brainmuseum.org) [192], BrainMaps.org [193], the Scalable Brain Atlas [194], the Digital Brain Bank [195], and the Animal Brain Collection [196]—which cover many mammalian species and, in the last case, other vertebrates. Domestic and large mammals, with their distinct evolutionary histories, sensory specializations, and spontaneous diseases, are therefore an undervalued source of comparative neuroanatomical data [24,25,197,198,199]. Available atlases and reference studies in species of veterinary interest are listed in Table S1 (Supplementary Materials) and summarized in Figure 2.

## 7. Clinical and Diagnostic Implications of Comparative Brain Mapping

Neurohistological analysis and comparative brain mapping contribute to veterinary neurological diagnosis by providing information on tissue organization, lesion distribution, cellular pathology, and species-specific anatomical variation. Neurological examination and magnetic resonance imaging (MRI) are fundamental to the investigation of brain disorders, but imaging findings do not always establish the cause of disease. Histopathology, immunohistochemistry, and molecular analyses complement this information; integrating them with clinical findings and neuroanatomical localization is essential for interpreting brain lesions [200,201].

### 7.1. Differential Diagnosis of Inflammatory, Degenerative, and Vascular Lesions

Histological examination, using hematoxylin and eosin staining and immunohistochemistry where indicated, enables the characterization of tissue damage and cellular responses and, together with clinical and MRI findings, supports the distinction between inflammatory, degenerative, and vascular processes [24,26,28,30]. Wolff et al. [201] conducted a multicenter retrospective study of 121 dogs: 77 had histologically confirmed brain disease, while 44 had a clinical diagnosis of idiopathic epilepsy. Overall, MRI detected the presence of a brain lesion with a sensitivity of 94.4% and a specificity of 95.5%. However, sensitivity for classifying cerebrovascular disease was 38.9% when images were interpreted without access to clinical data. This estimate was based on six dogs and had a wide 95% confidence interval (16.1–67.0%); it should therefore be interpreted cautiously and understood as referring to the classification of the cerebrovascular disease category in this study population.

Necrotizing meningoencephalitis, necrotizing leukoencephalitis, and granulomatous meningoencephalomyelitis are commonly grouped under the term meningoencephalitis of unknown origin (MUO). A presumptive diagnosis is based on neurological examination, MRI, cerebrospinal fluid analysis, and the exclusion of infectious causes. Definitive subtype classification, however, requires histopathological examination, generally performed post mortem or, less frequently, on an ante-mortem biopsy [202,203,204,205]. The limited availability of ante-mortem histopathological confirmation also affects treatment decisions. Available reviews primarily describe immunosuppressive treatments, but they draw on heterogeneous studies and do not establish a clearly superior treatment protocol [202,203]. Histopathological examination allows the disease subtype to be defined, but its value in guiding treatment selection and predicting outcomes remains unclear. In vascular lesions, histopathology likewise enables the characterization of ischemic damage and hemorrhage; its contribution nevertheless depends on lesion location, disease stage, sampling, and tissue preservation.

### 7.2. Distinguishing Normal Age-Related Changes from Pathological Alterations

Comparative brain mapping can help distinguish physiological aging from pathological changes, particularly in companion animals with progressive behavioral or cognitive decline. Borràs et al. [206] compared the brains of 20 aged dogs and 10 young dogs, documenting age-related changes in the meninges, choroid plexuses, blood vessels, neurons, and glial cells. These included lipofuscin accumulation, polyglucosan bodies, cerebrovascular amyloid deposition, and ubiquitin-positive structures; some of these changes may contribute to canine cognitive dysfunction.

On MRI, age-associated changes include iron-related hypointensities in the globus pallidus and substantia nigra [207] (Section 7.3), as well as cerebral atrophy. Pugliese et al. [208] assessed cerebral atrophy using a semiquantitative rating scale in 98 dogs representing 27 breeds and identified differences associated with skull shape. Youssef et al. [29] reviewed age-related neuropathological changes in domestic and laboratory animals; comparative studies of aging and naturally occurring diseases in veterinary species [24,25,199] further demonstrate the value of species-specific reference data. Atrophy, iron accumulation, or amyloid deposition should therefore not automatically be interpreted as evidence of a specific neurological disease, but should instead be evaluated in relation to age, clinical findings, and, when available, pathological confirmation.

### 7.3. Correlation Between MRI Findings and Microscopic Tissue Organization

MRI provides noninvasive information on brain morphology, whereas histology enables the examination of cellular and microstructural features that cannot be resolved by conventional MRI. In a study of 19 Beagle dogs aged 8 months to 16 years, Kimotsuki et al. [207] observed a correlation between age and lateral ventricular space volume, as well as age-associated hypointensities in the globus pallidus and substantia nigra on T2-weighted images. In the single 16-year-old Beagle that underwent post-mortem examination, Perls’ staining revealed iron accumulation in areas corresponding to the MRI hypointensities. The same animal also had a hyperintense area in the periventricular white matter, which the authors interpreted as a possible perivascular alteration, although this finding was not specifically verified histologically. Advanced MRI approaches, including measurements of the apparent diffusion coefficient and magnetization transfer ratio, as well as magnetic resonance spectroscopy, can provide complementary information on tissue composition. However, the correspondence between these parameters and specific histopathological processes requires further validation, ideally through studies correlating MRI with post-mortem histology, supported by the atlases and registration systems described in Section 6 and Section 8.

### 7.4. Identification of Cellular and Molecular Alterations in Naturally Occurring Neurological Diseases

Immunohistochemistry and molecular analyses can document cellular and molecular alterations that are not detectable by conventional MRI. In their study of canine cognitive dysfunction, Schütt et al. [30] examined the prefrontal cortex of 15 aged dogs with normal cognition, mild cognitive impairment, or cognitive dysfunction, comparing it with that of two young dogs. They detected diffuse cortical amyloid-β deposits and accumulation of the peptide in vessel walls. Amyloid-β pathology correlated with age but not with the severity of cognitive impairment; no neurofibrillary tau pathology was found, and proinflammatory cytokine levels were generally low, with no significant associations with cognitive status. Extending these analyses to other naturally occurring diseases requires species-specific validation of the antibodies and markers used (Section 3).

### 7.5. Diagnostic Models and Prognostic Assessment

Digital histology and artificial intelligence could standardize lesion quantification and reduce observer-related variability, for example when measuring neuronal density, inflammatory cell burden, gliosis, and tissue loss. Open resources such as CISCA and CytoDArk0 [100] provide a methodological basis for automated cell segmentation in Nissl-stained brain sections. However, their diagnostic or prognostic value in veterinary patients has not yet been demonstrated; evaluating this value will require disease-specific datasets, validated annotations, and testing across institutions and acquisition protocols. Cell segmentation, cell-type classification, density estimation, and unbiased estimation of total cell number are distinct tasks; the last requires design-based stereological sampling, and all of them require validation against histological ground truth by experts.

Overall, the established clinical applications of neurohistology include the characterization of brain lesions, the interpretation of abnormalities observed on imaging, and the study of brain aging. Automated multimodal diagnosis and prognostic assessment remain promising but have not yet been validated.

## 8. Current Challenges and Future Directions

As noted in the Introduction, most large research infrastructures, standard brain atlases, curated image repositories, and machine learning pipelines have been developed for humans, non-human primates, and mice, where large standardized datasets allowed robust methods to be built [14,15,209]. Veterinary neuroscience is therefore underrepresented in large computational and neuroinformatic initiatives.

Digital pathology has developed rapidly over the past decade [22,150,210], and high-throughput whole-brain histological pipelines have already been applied to the mouse brain [174]. Their implications for veterinary neurological diagnosis are discussed in Section 7.

### 8.1. Anatomical Variability and the Transfer of Human, Mouse, and Rat Models

A major obstacle is the large anatomical variability among mammalian brains.

Differences in cortical layers, white matter architecture, and regional proportions make it difficult to transfer processing pipelines developed for human, mouse, or rat brains [142,197].

This variation can reduce both the accuracy and the biological interpretability of computational methods applied to species with very different brain organization [17,211]. Model performance should therefore be reported separately for internal test sets, independent laboratories, and new species, and differences in staining and scanners, fixation, annotation uncertainty, and phylogenetic differences in brain organization should be treated as distinct sources of error.

### 8.2. Data Fragmentation, Standardization, and Annotation

Comparative analyses are also limited by differences in tissue preparation, image acquisition, processing, and annotation. Existing resources are spread across institutions, produced with different methods, and often lack standard metadata and compatible annotations [122]. Experience in neuroinformatics shows that poorly organized data and metadata make data sharing, reuse, and automated processing much more difficult [123].

Many veterinary species still lack standard stereotaxic coordinates and whole-brain atlases. Published atlases and volumetric studies rarely cover the whole brain and use different acquisition protocols, which makes it difficult to compare data between institutions and species (Table S1). The available evidence for each species, including brain regions, methods, sample types, main findings, and limitations, is summarized in Table S2 (Supplementary Materials), which also distinguishes methods directly applied to veterinary species from those whose application is still potential.

Some historical comparative collections are difficult to use in current computational workflows because their digitization, annotation, metadata, and access differ. The Comparative Mammalian Brain Collection (formerly neurosciencelibrary.org, now brainmuseum.org), for example, offers extensive histological material from many mammalian species [192], but its digital coverage, annotation, and metadata vary widely between specimens. Other repositories focus on a single type of data (e.g., braincatalogue.org), which limits the integration of histological, MRI, molecular, and connectivity data. These limitations reduce the reproducibility and scalability of comparative research. Direct evidence from studies in veterinary species is summarized in Table 4, and Table 5 compares the main methods, their advantages and limitations, and their applications in veterinary species, to help choose the most suitable method for a given question.

**Table 4:** Direct evidence from neurohistological, morphometric, imaging, and neuropathological studies in veterinary species. For each study, the table reports the species, the brain region investigated, the method, the sample type, the objective, the main findings, the limitations, and the reference. Only studies in which the method was applied to the species indicated are included; approaches whose application to veterinary species remains potential are discussed in the text and in Table S2. Abbreviations: DTI, diffusion tensor imaging; GME, granulomatous meningoencephalomyelitis; MRI, magnetic resonance imaging; NME, necrotizing meningoencephalitis; V1, primary visual cortex.

Species	Brain Region	Method	Sample Type	Objective	Main Findings	Limitations	References
Horse (*Equus caballus*)	Cerebral cortex	Nissl and myelin staining; cytoarchitectonic classification	Post-mortem brains from two adult horses	Classify cortical areas according to cellular and laminar organization	Five cortical types distinguished on the basis of cytoarchitectonic characteristics	Small sample; morphological classification does not validate functional boundaries	[89]
Horse (*Equus caballus*)	Motor cortex	Nissl staining; cytoarchitectural and laminar analysis compared with chimpanzee and macaque	Nissl-stained motor cortex sections from horse, chimpanzee, and macaque	Characterize cortical organization in a monodactylous mammal	Differences in laminar organization relative to primates, highlighting the limits of transferring canonical cortical models across species	Limited cortical coverage; comparability across studies	[50]
Sheep (*Ovis aries*)	Motor cortex	Laminar cytoarchitectonic analysis, description of cortical projections, and diffusion tensor imaging	Histological material and ex vivo imaging data	Describe laminar organization and the intracranial pyramidal and extrapyramidal tracts	Integrated description of cortical lamination and descending motor pathways	Diffusion imaging does not resolve cellular connections; limited sample size	[73]
Sheep (*Ovis aries*)	Orbitofrontal cortex	Nissl histology, neurochemistry, and diffusion tensor imaging (DTI); comparison with chimpanzee and human	Histological samples (cytoarchitecture, neurochemistry) and DTI samples, analyzed separately	Characterize topography, lamination, neurochemistry and connectivity	Species-specific cytoarchitectural and neurochemical features investigated alongside MRI-derived connectivity	Registration, sampling and cross-modal comparability may limit generalization	[105]
Sheep (*Ovis aries*)	Whole brain and major structures	Design-based stereology (Cavalieri estimator, point counting) on Mulligan-stained brain slabs	Five formalin-fixed brains, 10–12 slabs per brain	Estimate total brain volume and the volumes of major structures	Volumes and relative proportions of grey matter, white matter and selected structures quantified	Small sample; manual quantification	[108]
Cattle (*Bos taurus*)	Cerebellum	Digitized Nissl histology; multivariate morphometric and statistical modeling	Brains of male, female and intersex (freemartin) individuals	Quantify sex-associated differences in cerebellar cytoarchitecture	Sex-associated differences in cerebellar cytoarchitecture identified	Restricted to selected cerebellar regions; dependence on sampling and biological covariates	[166]
Ungulates	Primary visual cortex	Cytoarchitectonic analysis; comparison across ungulates and with primates	Post-mortem brain tissue	Characterize the organization of the primary visual cortex	Comparative description of V1 cytoarchitecture across ungulate species	Limited specimen availability and heterogeneous post-mortem histories	[18]
Pig and wild boar (*Sus scrofa*)	Primary motor cortex	Comparative cytoarchitectural and proteomic analysis	Brain tissue from domestic pigs and wild boars	Investigate the effects of domestication on cortical organization	Domestication-associated differences between domestic pigs and wild boars	Limited reference data restrict cross-study comparison	[169]
Dog (*Canis lupus familiaris*)	Whole brain	Histology-based stereotaxic atlas	Beagle brain sections	Provide stereotaxic coordinates for the canine brain	Stereotaxic reference for anatomical localization in the Beagle	Single breed; no population variability	[171]
Dog (*Canis lupus familiaris*)	Cerebral cortex	MRI-based stereotaxic cortical atlas	MRI data	Support standardized anatomical localization	Species-specific MRI cortical reference	Does not establish histological boundaries or validate automated segmentation	[185]
Dog (*Canis lupus familiaris*)	Whole brain	MRI-based morphometric network analysis	Clinical MRI data	Investigate associations between brain morphometry, hormonal status and disease risk	Brain morphometry linked tumour risk to oestrogen deficiency and accelerated brain ageing	Retrospective imaging data; no histological validation	[168]
Cat (*Felis catus*)	Cerebral cortex	High-field MRI; cortical atlas and tissue probability maps	T1-weighted 7-T MRI from eight adult cats	Establish a standardized three-dimensional cortical reference space	Seventy-one cortical areas mapped onto an averaged MRI template	MRI-based; does not validate cell-level segmentation in histological sections	[175]
Horse (*Equus caballus*)	Whole brain	MRI-based anatomical characterization	MRI data	Describe brain anatomy and provide imaging reference information	Anatomical imaging references for a large domestic mammal	Protocols and anatomical coverage differ among studies	[186,187]
Pig (*Sus scrofa domesticus*)	Whole brain, cerebral cortex	Integrative MRI-based anatomical study	MRI data	Establish an anatomical reference for the porcine brain, with emphasis on cortical external morphology	Species-specific framework for anatomical localization	No cellular-level validation	[188]
Dog (*Canis lupus familiaris*)	Whole brain	MRI compared with histologically confirmed diagnoses	121 dogs	Evaluate MRI differentiation of neoplastic, inflammatory and cerebrovascular disease	High sensitivity for lesion detection; low sensitivity (38.9%) for classifying cerebrovascular disease	Retrospective design; performance varies with disease category	[201]
Dog (*Canis lupus familiaris*)	Brain lesions in NME and GME	Histopathology and immunohistochemistry	Brain tissue from affected dogs	Characterize the pathological and immunological features of necrotizing and granulomatous meningoencephalitis	Distinct pathological and immunological features supporting disease classification	Post-mortem material; limited ante-mortem applicability	[204]
Dog (*Canis lupus familiaris*)	Whole brain	Routine and special stains; immunohistochemistry	Brains of old and young dogs	Characterize age-related changes in the canine brain	Lipofuscin, polyglucosan bodies, cerebrovascular amyloid and ubiquitin-positive structures in aged dogs	Age-related changes not directly related to clinical severity	[206]
Dog (*Canis lupus familiaris*)	Globus pallidus, substantia nigra, ventricles	MRI with Perls’ staining	19 Beagle dogs aged 8 months to 16 years	Describe age-related MRI changes and their tissue correlates	T2-weighted hypointensities corresponded to iron accumulation	Single breed; small sample; histological confirmation in a single dog	[207]
Dog (*Canis lupus familiaris*)	Prefrontal cortex	Neuropathological and inflammatory marker analysis	15 aged dogs with normal cognition, mild cognitive impairment or cognitive dysfunction; two young dogs as controls	Relate cognitive status to tissue pathology	Amyloid-β deposits correlated with age but not with severity of cognitive impairment; no neurofibrillary tau pathology; low proinflammatory cytokine levels	Small sample; relationships with clinical severity and progression require further study	[30]

**Table 5:** Comparative overview of neurohistological, imaging, and computational methods for neuroanatomical analysis in veterinary species. For each method, the table reports the type of information provided, the main advantage, the key limitation, and the current veterinary application, to support the selection of the most appropriate method for a given neuroanatomical or diagnostic question. In the last column, “Direct” indicates a documented application in veterinary species; “Limited” a documented application subject to specific practical or technical constraints; and “Potential” an application not yet adequately validated in the veterinary context considered. A method may have been applied directly in one species or context while remaining potential in another. Abbreviations: MRI, magnetic resonance imaging.

Method	Type of Information	Main Advantage	Key Limitation	Veterinary Application
Nissl staining and cytoarchitectonic analysis	Neuronal and glial density, cell size, laminar and areal organization	Simple and robust; applicable to post-mortem and archival tissue of any species	No information on connectivity or neurochemical phenotype; two-dimensional sampling	Direct: cortical and cerebellar cytoarchitecture in horse, sheep and cattle [50,166,170]
Golgi and Golgi–Cox impregnation	Dendritic and spine morphology of individual neurons	Unique single-neuron morphological detail	Unpredictable and incomplete labeling; sensitive to fixation delay; laborious quantification	Potential: no veterinary application is reviewed here; feasibility depends on post-mortem interval and fixation quality [42,43]
Myelin and silver staining	Myeloarchitecture, fiber systems and neurofibrils	Low cost; complements Nissl staining for areal delineation	Largely qualitative; no information on the direction of connections	Direct: cytoarchitectonic and myeloarchitectonic classification of the horse cerebral cortex [46,47,48,89]
Histochemistry and immunohistochemistry	Neurochemical phenotype, cell types, glial reactivity and disease-associated proteins	Cellular and molecular specificity in situ	Antibody cross-reactivity and specificity must be validated for each species; mostly semi-quantitative	Direct: neurochemical characterization of sheep motor and orbitofrontal cortex [73,105]; veterinary neuropathology (Section 7)
Axonal tract tracing	Point-to-point connectivity and direction of projections	Reference standard for the demonstration of neural connections	In vivo tracer injection is now rarely feasible in veterinary species; post-mortem lipophilic dyes trace only short distances	Limited: documented in sheep, dogs, cats and rabbits [67,68,69,70,71,72], but in vivo tracer injection is now rarely feasible; post-mortem lipophilic dyes trace only short distances [63]
Design-based stereology	Unbiased estimates of cell number, volume and length	Statistically unbiased and efficient quantification	Labor-intensive; systematic sampling of entire structures is difficult in large brains	Direct: volumetric estimation in the brains of sheep, goats, and rabbits [7,8,108,109,110]
Structural MRI and MRI-based atlases	Gross morphology, volumes and stereotaxic reference templates	Non-invasive, whole-brain and clinically available	No cellular resolution; species-specific templates and protocols are required	Direct: atlases and reference studies in dog, cat, horse and pig [175,185,186,187,188]; clinical diagnosis (Section 7)
Diffusion MRI and tractography	White-matter microstructure and large-scale connectivity	Whole-brain connectivity, also applicable ex vivo to post-mortem brains	Indirect measure; false-positive and false-negative reconstructions; no directionality	Direct: ex vivo diffusion imaging of large animal brains, including sheep [73,74,105]
Tissue clearing and light-sheet microscopy	Three-dimensional cellular architecture of intact tissue	Cellular resolution in three dimensions without physical sectioning	Limited sample size; slow clearing and antibody penetration in large brains	Potential: developed mainly in mice; applicable to small tissue blocks of larger species [82,83,84,85,86]
Whole-slide imaging and digital pathology	Digitized histological sections for quantitative analysis	Archiving, sharing and reproducible measurement	Variability in staining and scanning; data storage and management requirements	Direct in veterinary diagnostic pathology [22]; potential in veterinary neuroanatomical research
Deep-learning cell segmentation and classification	Automated cell detection, classification and morphometry	Scalable and reproducible quantification of large image collections	Requires representative annotations; sensitive to domain shift; limited interpretability	Potential: applied to Nissl-stained mammalian brain tissue [100,104]; validation in individual veterinary species is still required (Table S2)
Multimodal registration and molecular atlases	Integration of histology, MRI, connectivity and gene expression in common coordinate systems	Cross-scale and cross-modal integration	Registration errors; heterogeneous metadata; few veterinary reference spaces	Potential: developed mainly for human and mouse brains [14,15,16,173]; requires interoperable infrastructures [122,123]

### 8.3. Future Directions

Artificial intelligence offers important opportunities to address these limitations. Automated segmentation, morphometry, and multimodal registration can reduce manual work and allow the analysis of large image collections [38,212,213]. These tools could speed up cytoarchitectural mapping in large-brained species, such as cattle and horses, for which manual analysis is very time-consuming. Self-supervised learning, weak supervision, and transfer learning may partly overcome the lack of annotations and improve performance across datasets [11,132].

Future progress will depend on three complementary directions: AI-assisted tissue analysis, multimodal integration of imaging and molecular data, and explicitly comparative frameworks across species.

A priority is the development of multimodal atlases that combine cytoarchitecture, MRI, connectivity, and molecular data in shared or linked coordinate systems [16,122,214].

This will require open data infrastructures, common metadata standards, shared annotation protocols, and reference frameworks across species. For veterinary neuroscience, the challenge is not simply to reuse tools developed for humans or laboratory animals, but to develop methods that allow standardized quantitative comparison while preserving differences between species, because subtle structural differences may reflect real evolutionary divergence rather than technical variation to be removed during image registration or model training.

## 9. Conclusions

This review shows that the analysis of the brain has developed through cumulative rather than substitutive methodological advances. Gross anatomical dissection and classical preparations, such as Nissl and Golgi staining, remain the material on which macroscopic and microscopic morphometry, stereology, digital histology, and deep learning now operate, and each step has added a new level of information, from brain volume and white matter organization to cell morphology, neurochemistry, connectivity, and quantitative cytoarchitecture. In domestic animals, however, direct evidence is still limited to a few species, mainly the dog, horse, sheep, cattle, pig, and cat, and to selected brain regions, while most computational tools and multimodal atlases developed in humans and laboratory animals remain potential rather than validated applications.

Three main implications follow. First, reference brain atlases and quantitative normative data for each domestic species are a priority for veterinary neurology and neuropathology, because the interpretation of brain lesions depends on them. Second, methods developed in humans, mice, and rats should be validated on the brains of domestic animals before use, and their outputs should remain interpretable in anatomical terms. Third, the open sharing of brains and digital data from domestic animals, including animals examined at necropsy, would allow current analytical frameworks to be tested across the brains of different species.

## Figures and Tables

**Table 3:** Overview of image analysis approaches applied to histological material, from early digitization and rule-based methods to machine learning and deep learning paradigms, and multimodal integration, with relevance for veterinary neuroanatomy. For each approach, paradigm, or cross-cutting issue, the table reports the specific methods or architectures employed, the main methodological contribution and advantages, the key limitation, and the relevance for veterinary neuroanatomy. The final two rows highlight cross-cutting issues relevant across paradigms: interpretability limitations associated with deep learning models, and domain shift and annotation scarcity as major obstacles to the application of these methods to veterinary and comparative neuroscience datasets.

Paradigm/Challenge	Methods/Architectures	Main Contribution and Advantages	Key Limitation	Veterinary Neuroanatomy Relevance	References
Transition from analog microscopy to digital imaging	Photomicrography and early digitization of histological sections	Transformed histological observations into persistent, reproducible, and potentially computable image data	Limited resolution, standardization, storage capacity, and analytical capabilities	Established the basis for quantitative analysis and digital archiving of veterinary brain histology	[133,134]
Early computational morphometry	Handcrafted feature extraction based on geometry, intensity, texture, and spatial relationships	Introduced quantitative descriptors of cellular and tissue organization	Observer-dependent feature selection; sensitivity to imaging and staining conditions	Enabled quantitative characterization but remained difficult to generalize across species and protocols	[119,120]
Rule-based image analysis	Thresholding, region-based methods, template matching, and rule-based segmentation	Enabled partial automation of cellular and structural identification	Sensitivity to noise, staining variability, cell clustering, and parameter selection	Limited robustness in heterogeneous histological material and across veterinary species	[135,136]
Classical machine learning	Support Vector Machines, Random Forests	Improved classification and segmentation using statistical learning on handcrafted features	Strong dependence on engineered descriptors; performance depends on the suitability of handcrafted features	Performance may be sensitive to interspecies differences in morphology and staining; requires validation in the target species	[137,138]
Early biomedical image analysis	Feature-based tissue and cell classification/segmentation pipelines	Increased automation of histological image analysis	Limited representation capacity; sensitivity to feature-design bias	Sensitive to staining, acquisition, and tissue-preparation variability in veterinary datasets	[11,139,140]
Deep learning (CNNs)	Convolutional neural networks	End-to-end hierarchical feature learning directly from image data	Reduced interpretability; high dependence on representative training data	Supervised training requires representative annotations; limited datasets can constrain applications in veterinary neurohistology	[10,100,126]
Biomedical segmentation models	U-Net, 3D U-Net, nnU-Net	Enabled automated segmentation of histological and imaging-derived structures	Sensitive to domain shift, dataset bias, and differences between training and target data	Generalization across species, staining protocols, and acquisition settings requires independent validation	[99,127,141]
Transformer-based models	Vision Transformers and attention-based architectures	Attention-based modeling of relationships across image patches	High data and computational requirements; limited biological interpretability	Potential for capturing broader cytoarchitectural context; requires validation on veterinary neurohistological datasets	[128,129]
Multimodal and comparative neuroinformatics	Image registration, multimodal integration, and standardized coordinate systems	Integration of histology, MRI, connectivity, and molecular information across spatial scales	Registration errors, heterogeneous acquisition protocols, incomplete metadata, and interspecies anatomical variability	Major requirement for interoperable comparative atlases and cross-species neuroanatomical analysis	[16,122,123]
Key cross-cutting challenge	Interpretability of learned representations	Predictive performance does not necessarily establish biological interpretability	Limited transparency of learned features and decision processes	Critical when computational outputs are used to infer anatomical or cytoarchitectural organization	[130,131,132]
Veterinary-specific challenge	Domain shift, anatomical heterogeneity, and annotation scarcity	Highlights the translational gap between datasets generated in model species and veterinary datasets	Poor cross-domain generalization; limited labeled data	Major bottlenecks for comparative and veterinary neuroanatomical applications	[17,22,100,142]

## Data Availability

No new data were created or analyzed in this study. Data sharing is not applicable to this article.

## Supplementary Materials

The following supporting information can be downloaded at: https://www.mdpi.com/article/10.3390/ani16213486/s1, Table S1: Brain atlases and reference studies across human, laboratory, and veterinary species; Table S2: Representative studies on the brains of domestic species and computational approaches for cytoarchitectural analysis, distinguishing direct from potential applications; Table S3: Search strings used in PubMed/MEDLINE, Scopus, and Web of Science Core Collection. References [215,216,217,218,219,220,221,222,223,224,225,226,227,228,229,230,231,232,233,234,235,236,237,238,239,240,241,242,243,244,245,246,247,248,249,250,251,252,253,254,255,256,257,258,259,260,261,262,263,264,265,266,267,268,269,270,271,272,273,274,275,276,277,278,279,280,281,282,283,284,285,286,287,288,289,290] are cited in the Supplementary Materials.
