# Supplementary material for: From Silver Stains to Deep Learning: Neurohistological Analysis and Digital Mapping of the Brain of Domestic and Laboratory Animals in Veterinary Neuroscience
Source: Animals (Basel). 2026 Nov 4;16(21):3486. doi: 10.3390/ani16213486 (PMC13762009; doi:10.3390/ani16213486)
Supplement: Supplementary file 1 [file animals-16-03486-s001.zip › animals-4628635-supplementary.pdf]

Table S1. Brain atlases and reference studies across human, laboratory, and veterinary species.

SPECIES	ATLAS NAME	PAPER	TECHNIQUE	YEAR	REFERENCE
Felis catus	Magnetic resonance imaging of the normal feline brain		MRI atlas	1995	[215]
Felis catus		Investigation of feline brain anatomy for the detection of cortical spreading depression with magnetic resonance imaging	MRI anatomy	2001	[216]
Felis catus	Normal feline brain: clinical anatomy using magnetic resonance imaging		MRI (clinical) atlas	2011	[190]
Felis catus	High resolution MRI anatomy of the cat brain at 3 Tesla		MRI anatomy	2014	[217]
Felis catus		Comparison of feline brain anatomy in 0.25 and 3 Tesla magnetic resonance images	MRI anatomy	2017	[218]
Felis catus	A neuroanatomical study of the feline brain using MRI and Mulligan staining: functional and pathological considerations		MRI and macroscopic anatomy	2021	[219]
Felis catus	Catlas: A magnetic resonance imaging-based three-dimensional cortical atlas and tissue probability maps for the domestic cat (Felis catus)		MRI atlas	2017	[175]
Felis catus	Stereotaxic diffusion tensor imaging white matter atlas for the in vivo domestic feline brain		MRI/DWI atlas	2020	[220]
Felis catus	The brain stem of the cat; a cytoarchitectonic atlas with stereotaxic coordinates		Stereotaxic histology, Nissl staining	1968	[221]
Felis catus	A stereotaxic atlas of the prefrontal cortex of the cat		Stereotaxic histology, Nissl staining	1977	[222]
Felis catus	A stereotaxic atlas of the cat brain		Stereotaxic histology, Nissl staining	1961	[223]
Felis catus	Stereotaxic atlases for the cat, rabbit and rat		Stereotaxic histology, Nissl staining	1967	[224]
Canis lupus familiaris	A stereotaxic atlas of the dog's brain		Stereotaxic histology, Nissl staining	1960	[225]
Canis lupus familiaris	The Beagle Brain in Stereotaxic Coordinates		Stereotaxic histology	2011	[171]
Canis lupus familiaris	A digital atlas of the dog brain		MRI atlas	2012	[226]
Canis lupus familiaris	A detailed canine brain label map for neuroimaging analysis		MRI atlas	2019	[227]
Canis lupus familiaris	A stereotaxic breed-averaged, symmetric T2w canine brain atlas		MRI atlas	2019	[228]

Canis lupus familiaris	Stereotaxic cortical atlas of the domestic canine brain		MRI atlas	2020	[185]
Canis lupus familiaris	A high-resolution MRI brain template for adult Beagle		MRI atlas	2020	[229]
Canis lupus familiaris		Magnetic resonance imaging of the canine brain at 7 T	MRI anatomy	2009	[230]
Canis lupus familiaris		Clinical anatomy of the canine brain using magnetic resonance imaging	MRI clinical anatomy	2007	[231]
Canis lupus familiaris		Magnetic resonance imaging of the canine brain at 3 and 7 T	MRI anatomy	2011	[232]
Canis lupus familiaris		Normal canine brain: comparison between magnetic resonance imaging and cross-sectional anatomy	MRI and macroscopic anatomy	2011	[233]
Canis lupus familiaris		Significant neuroanatomical variation among domestic dog breeds	MRI across different breeds	2019	[234]
Canis lupus familiaris		Ex vivo susceptibility-weighted imaging anatomy of canine brain–comparison of imaging and histological sections	MRI and macroscopic anatomy	2022	[235]
Canis lupus familiaris		T2-weighted turbo spin-echo magnetic resonance imaging of canine brain anatomy at 1.5T, 3T, and 7T field strengths	MRI atlas	2022	[236]
Canis lupus familiaris		Characterization of structural connectivity of the default mode network in dogs using diffusion tensor imaging	MRI/DWI atlas	2016	[237]
Canis lupus familiaris		MRI, CT and high resolution macro-anatomical images with cryosectioning of a Beagle brain: Creating the base of a multimodal imaging atlas	MRI, CT, cryosection anatomy	2019	[238]
Canis lupus familiaris	The canine brain in stereotaxic coordinates	The canine brain in stereotaxic coordinates: full sections in frontal, sagittal, and horizontal planes	Stereotaxic histology, cytoarchitectonics	1970	[239]
Bos taurus	Neuroanatomy of the calf brain as revealed by high-resolution MRI		MRI anatomy	2009	[240]
Bos taurus	Brain atlas of cattle		Stereotaxic histology	2002	[241]
Capra hircus	Stereotaxic atlas of the goat brain for an accurate approach to the hypothalamic nuclei		Stereotaxic technique, histology	2015	[242]
Capra hircus	Atlas of the brains of domestic animals		Stereotaxic histology, Nissl staining	1968	[243]
Capra hircus		A study of the comparative anatomy of the brain of domestic ruminants using magnetic resonance imaging	MRI anatomy	2012	[244]
Capra hircus	A stereotaxic atlas of the medulla oblongata of the goat's brain		Stereotaxic histology	1987	[245]
Equus caballus		Magnetic resonance imaging of the brain of normal neonatal foals	MRI anatomy	1997	[246]

Equus caballus		Magnetic resonance imaging of the normal equine brain	MRI atlas	2001	[186]
Equus caballus		Magnetic resonance imaging of two normal equine brains and their associated structures	MRI anatomy	2001	[247]
Equus caballus		3-Tesla magnetic resonance imaging of the equine brain in healthy horses–Potentials and limitations	MRI anatomy	2014	[248]
Equus caballus		Neuroanatomy of the equine brain as revealed by high-field (3 Tesla) magnetic-resonance-imaging	MRI atlas	2019	[187]
Equus caballus	Equine stereotaxtic population average brain atlas with neuroanatomic correlation		MRI atlas	2019	[249]
Equus caballus	Atlas of the brains of domestic animals		Stereotaxic histology, Nissl staining	1968	[243]
Lama glama	Brain atlas of the llama (Lama glama)		Nissl	2001	[250]
Ovis aries		3 Tesla magnetic resonance imaging and multiplanar reconstruction of the brain and its associated structures in sheep	MRI anatomy	2020	[251]
Ovis aries	Construction of an MRI 3D high resolution sheep brain template		MRI atlas	2015	[252]
Ovis aries	Computation of a high-resolution MRI 3D stereotaxic atlas of the sheep brain		MRI atlas	2017	[253]
Ovis aries	A stereotaxic, population-averaged T1w ovine brain atlas including cerebral morphology and tissue volumes		MRI atlas	2015	[254]
Ovis aries		A study of the comparative anatomy of the brain of domestic ruminants using magnetic resonance imaging	MRI anatomy	2012	[244]
Ovis aries	Orbitofrontal cortex — regional cytoarchitectural dataset		Nissl histology, neurochemistry, DTI	2022	[105]
Ovis aries	Motor cortex — regional cytoarchitectural dataset		Nissl histology, immunohistochemistry, DTI	2019	[73]
Ovis aries	In vivo Diffusion Tensor Magnetic Resonance Tractography of the Sheep Brain: An Atlas of the Ovine White Matter Fiber Bundles		MRI, diffusion tractography	2019	[255]
Ovis aries	Atlas stéréotaxique du cerveau de Brebis “Préalpes-du-Sud”		Stereotaxic histology, Nissl staining	1967	[256]
Ovis aries	Atlas of the brains of domestic animals		Stereotaxic histology, Nissl staining	1968	[243]

Sus scrofa		Preliminary results of a magnetic resonance imaging (MRI) study of the pig brain placed in stereotaxic conditions	MRI atlas	1993	[257]
Sus scrofa		3 Tesla Magnetic resonance imaging and multiplanar reconstruction of the brain and its associated structures in pig	MRI anatomy	2011	[258]
Sus scrofa	A three-dimensional digital segmented and deformable brain atlas of the domestic pig		MRI atlas	2010	[259]
Sus scrofa	An in vivo three-dimensional magnetic resonance imaging-based averaged brain collection of the neonatal piglet (Sus scrofa)		MRI atlas	2014	[260]
Sus scrofa	Development of new population-averaged standard templates for spatial normalization and segmentation of MR images for postnatal piglet brains		MRI atlas	2014	[261]
Sus scrofa	Standard Brain Template and Multi-atlas Based Segmentation of tgHD Minipig Brain		MRI/DWI atlas	2016	[262]
Sus scrofa	An in vivo multi-modal structural template for neonatal piglets using high angular resolution and population-based whole-brain tractography		MRI/DWI atlas	2016	[263]
Sus scrofa		Automatic delineation of brain regions on MRI and PET images from the pig	MRI/PET atlas	2018	[264]
Sus scrofa	A pig white matter atlas and common connectivity space provide a roadmap for the introduction of a new animal model in translational neuroscience		MRI/DWI atlas	2020	[265]
Sus scrofa	In vivo Population Averaged Stereotaxic T2w MRI Brain Template for the Adult Yucatan Micropig		MRI atlas	2020	[266]
Sus scrofa	High-resolution magnetic resonance imaging-based atlases for the young and adolescent domesticated pig (Sus scrofa)		MRI atlas	2021	[267]
Sus scrofa	MRI brain templates of the male Yucatan minipig		MRI atlas	2021	[268]
Sus scrofa	Stereotaxic atlas of the pig brain		Stereotaxic histology, Nissl staining, cytoarchitectonics	1999	[269]
Sus scrofa	A three-dimensional digital segmented and deformable brain atlas of the domestic pig		MRI anatomy	2010	[259]
Sus scrofa	MR-Based Statistical Atlas of the Göttingen Minipig Brain		MRI atlas	2001	[270]

Oryctolagus cuniculus	Rabbit brain research. Volume 1. Atlas for stereotaxic brain research on the conscious rabbit	Stereotaxic histology	1961	[271]
Oryctolagus cuniculus	A new stereotaxic atlas of the rabbit brain	Stereotaxic histology	1981	[272]
Oryctolagus cuniculus	Atlas of the Rabbit Brain and Spinal Cord	Histological atlas	1986	[273]
Oryctolagus cuniculus	A magnetic resonance image-based atlas of the rabbit brain for automatic parcellation	MRI atlas	2013	[274]
Oryctolagus cuniculus	A 3D multimodal MRI stereotaxic atlas of the rabbit brain	Multimodal MRI atlas	2026	[275]
Oryctolagus cuniculus	Magnetic resonance imaging anatomy of the rabbit brain at 3 T	MRI anatomy	2015	[191]
Homo sapiens	A diffusion tensor imaging tractography atlas for virtual in vivo dissections	MRI, diffusion tractography	2008	[276]
Homo sapiens	Multimodal parcellation of human cerebral cortex	MRI, myelin mapping, fMRI	2016	[15]
Homo sapiens	Allen human brain transcriptome atlas	RNA microarray, ISH	2012	[173]
Homo sapiens	Human connectome project	MRI, diffusion tractography, fMRI	2013	[209]
Macaca mulatta	A combined MRI and histology atlas of the rhesus monkey brain in stereotaxic coordinates	MRI	2006	[277]
Macaca mulatta	The rhesus monkey brain in stereotaxic coordinates	Stereotaxic, histological atlas	2000	[278]
Macaca mulatta	A diffusion tensor MRI atlas of the postmortem rhesus macaque brain	MRI/DTI	2015	[279]
Macaca mulatta	Rhesus macaque brain atlas regions aligned to an MRI template	MRI	2019	[280]
Macaca mulatta	The subcortical atlas of the rhesus macaque (SARM) for neuroimaging	MRI	2021	[281]
Pan troglodytes	A stereotaxic atlas of the chimpanzee brain (Pan satyrus)	Stereotaxic histology, Nissl staining	1965	[282]
Pan troglodytes	Chimpanzee brain morphometry utilizing standardized MRI preprocessing and macroanatomical annotations	T1-weighted MRI	2020	[283]
Pan troglodytes	A comprehensive atlas of white matter tracts in the chimpanzee	Diffusion MRI / tractography	2020	[284]
Pan troglodytes	The Chimpanzee Brainnetome Atlas reveals distinct connectivity and gene expression profiles relative to humans	MRI	2025	[285]
Mus musculus	Allen Mouse Brain Atlas	ISH, Nissl histology	2007	[14]

Mus musculus	The Allen Mouse Brain Common Coordinate Framework: A 3D Reference Atlas	MRI, histology	2020	[16]
Mus musculus	The Mouse Brain in Stereotaxic Coordinates	Stereotaxic, histological atlas	2001	[286]
Mus musculus	High resolution three-dimensional brain atlas using an average magnetic resonance image of 40 adult C57Bl/6J mice	MRI-3D	2008	[287]
Rattus norvegicus	The Rat Brain in Stereotaxic Coordinates	Stereotaxic histology, Nissl staining	2013	[288]
Rattus norvegicus	Brain maps 4.0—structure of the rat brain: an open access atlas with global nervous system nomenclature ontology and flatmaps	Histological staining	2018	[289]
Rattus norvegicus	Waxholm space atlas of the Sprague Dawley rat brain	MRI + DTI	2014	[290]
Rattus norvegicus	Age-dependent changes in the brain of albino rats (Rattus norvegicus): A morphometric analysis and magnetic resonance imaging	MRI, morphometry	2024	[111]
Mesocricetus auratus	A stereotaxic atlas of the golden hamster brain	Stereotaxic histology	2001	[40]

Table S1: The table reports the species, the atlas name (for named atlases, either whole-brain or region-specific) or the title of the study (for studies providing reference-level anatomical data on the brain of that species), the primary technique, the year of publication, and the reference. Species of veterinary interest (carnivores, ungulates including giraffids and rhinoceroses, lagomorphs, and cetaceans) are listed first, followed by the reference species (humans, non-human primates, mice, rats, and Syrian hamsters). The same inclusion criteria were applied to all species. The table is representative rather than exhaustive and provides the data used to construct Figure 2 of the main article. While MRI-based atlases are available for several domestic species, histological and multimodal atlases remain scarce outside human and laboratory species, and non-conventional species are represented only by isolated studies. Reference numbers refer to the reference list of the main article. Abbreviations: CT, computed tomography; DTI, diffusion tensor imaging; DWI, diffusion-weighted imaging; fMRI, functional magnetic resonance imaging; ISH, in situ hybridization; MRI, magnetic resonance imaging; PET, positron emission tomography.

Table S2. Representative studies on the brains of domestic species and computational approaches for cytoarchitectural analysis, distinguishing direct from potential applications.

Species	Brain region	Method	Sample type	Objective	Main findings	Limitations	References
Bos taurus	Cerebellum, vermal lobules VIII and IX	Direct application: digitized histological sections, neuronal morphometry and multivariate statistical analysis.	Cerebellar histological sections from male, female and freemartin cattle.	To quantify sex-associated differences in cerebellar cytoarchitecture.	Differences in cellular morphology and cytoarchitectural organization were investigated across groups.	Restricted to selected cerebellar regions; morphometric analysis does not establish deep-learning segmentation performance.	[166]
Bos taurus*, *Capra hircus*, *Ovis aries	Whole brain, cerebral sulci and gyri	Direct application: T2-weighted MRI, high-resolution MRI and three-dimensional anatomical reconstruction.	MRI datasets from domestic ruminants.	To compare gross brain morphology and anatomical organization among domestic ruminants.	Comparative MRI facilitated identification of major anatomical structures and species-associated morphological differences.	MRI-based anatomy does not directly establish microscopic cytoarchitecture or cellular segmentation accuracy.	[244]
Sus scrofa domesticus	Whole brain and major cerebral structures	Direct application: MRI, three-dimensional segmentation and digital atlas construction with histological support.	Domestic pig brain; one hemisphere used for atlas construction, with symmetry-based reconstruction.	To develop a three-dimensional digital atlas for anatomical localization and neuroimaging.	A digital reference atlas was constructed, incorporating numerous cerebral and other anatomical structures.	Limited individual sampling; symmetry-based reconstruction may not capture natural hemispheric asymmetry or population variability.	[259]
Sus scrofa domesticus	Whole brain	Direct application: high-resolution MRI and developmental digital atlas construction.	MRI datasets from young and adolescent domestic pigs.	To provide age-specific anatomical reference atlases for developmental neuroimaging.	MRI-based atlases were developed to facilitate standardized anatomical localization at different developmental stages.	MRI-derived segmentation does not establish histological boundaries at cellular resolution; age and individual variability remain relevant.	[267]
Felis catus	Cerebral cortex	Direct application: high-field MRI, cortical atlas construction, nonlinear registration and tissue probability mapping.	T1-weighted 7-T MRI from eight adult domestic cats.	To establish a standardized three-dimensional cortical reference space.	Seventy-one cortical areas were mapped onto an averaged MRI template, with tissue probability maps generated for neuroimaging analyses.	The atlas is MRI-based and primarily cortical; it does not validate automated cell-instance segmentation in histological sections.	[175]
Canis lupus familiaris	Whole brain and cortical surface	Direct application: population-based MRI template, image registration and cortical surface reconstruction.	In vivo MRI from 15 mesaticephalic dogs, supplemented by an ex vivo MRI template.	To establish a standardized canine brain reference for anatomical localization and group-level analyses.	A population-based digital atlas was developed to support quantitative canine neuroimaging.	Limited breed representation; MRI registration is not equivalent to histological segmentation or cell-level validation.	[226]
Capra hircus	Whole brain, cerebral sulci and gyri	Direct application: comparative MRI and three-dimensional anatomical reconstruction.	Goat brain MRI included in a comparative study of domestic ruminants.	To characterize gross brain anatomy and compare structural organization among ruminants.	Major anatomical structures and cortical folding patterns were examined using MRI.	The study focused on gross anatomy and did not validate digital histological segmentation or deep-learning algorithms.	[244]

Species	Brain region	Method	Sample type	Objective	Main findings	Limitations	References
Equus caballus	Cerebral cortex	Direct application: Nissl and myelin staining and cytoarchitectonic classification.	Post-mortem brains from two adult horses.	To classify cortical areas according to their cellular and laminar organization.	Five cortical types were distinguished on the basis of cytoarchitectonic characteristics.	Small sample; morphological classification does not independently validate functional boundaries or automated segmentation.	[89]
Equus caballus	Motor cortex	Direct application: digitized Nissl histology, cellular morphometry and comparative statistical analysis.	Histological material from horse, chimpanzee and macaque.	To compare laminar organization and neuronal morphology across species.	Differences in cortical lamination and neuronal morphology were characterized in the species examined.	Limited cortical coverage; quantitative morphometry does not constitute automated cell-instance segmentation.	[50]
Ovis aries	Orbitofrontal cortex	Direct application: Nissl histology, immunocytochemistry and diffusion tensor imaging.	Sheep brain tissue and imaging data.	To characterize cortical lamination, neurochemical organization and white-matter connectivity.	Species-specific cytoarchitectural and neurochemical characteristics were investigated alongside MRI-derived connectivity.	Limited sample sizes and regional coverage; diffusion imaging does not directly resolve cellular connections.	[105]
Ovis aries	Whole brain and major cerebral structures	Direct application: design-based stereology, Cavalieri estimator and point counting.	Five formalin-fixed sheep brains.	To estimate total brain volume and the relative volumes of major brain structures.	Brain volume and the relative proportions of grey matter, white matter and selected structures were quantified.	Small sample; stereological quantification does not establish automated histological segmentation.	[108]
Bos taurus, Tursiops truncatus, Mus musculus, Pan troglodytes, Macaca fascicularis.	Nissl-stained brain sections	Direct computational application: CISCA cell-instance segmentation and classification; CytoDArk0 annotated dataset. Potential application: extension to veterinary species not independently represented or validated.	Digitized histological images; approximately 40,000 annotated cells reported for the dataset.	To automate cellular segmentation and classification and provide an open dataset for cytoarchitectural analysis.	The study introduced a computational framework and annotated histological images for cellular analysis.	The species composition, per-species sample sizes and independent validation in *Bos taurus*, *Sus scrofa*, *Felis catus*, *Canis lupus familiaris*, *Capra hircus*, *Equus caballus* and *Ovis aries* must be verified before claiming direct applicability to each species.	[100]
Bos taurus*, *Sus scrofa*, *Felis catus*, *Canis lupus familiaris*, *Capra hircus*, *Equus caballus* and *Ovis aries	Species-specific cortical and subcortical regions, to be determined for each dataset	Potential application: transfer learning, domain adaptation, self-supervised learning, weak supervision and deep-learning-based cell-instance segmentation. These methods should be classified as direct applications only where the target species has actually been evaluated.	Veterinary histological images with appropriate reference annotations; a validated sample is not specified here.	To reduce annotation requirements and support scalable, quantitative comparisons across veterinary species.	These approaches may improve model adaptation to heterogeneous tissue and staining conditions, but their effectiveness in each species remains to be established.	Performance cannot be inferred from human, mouse, or rat datasets alone. Independent validation, representative annotations and assessment of biological interpretability are required.	[11,132]

Table S2: The table distinguishes methods directly applied to veterinary brain material from computational approaches whose use in the target species remains potential or insufficiently validated. Although anatomical

MRI, stereology, histology and digital atlases are available for several domestic mammals, these resources do not necessarily demonstrate the validity of automated cell segmentation or deep-learning models in those species. In particular, the availability of an MRI atlas should not be interpreted as evidence of a validated histological segmentation pipeline. For each study, the table reports the species, the brain region investigated, the method, the sample type, the objective, the main findings, the limitations, and the reference. Methods are classified as direct applications when the species was examined in the cited study, and as potential applications when their use in veterinary species has not yet been validated. Reference numbers refer to the reference list of the main article. Abbreviations: CISCA, cell instance segmentation and classification; MRI, magnetic resonance imaging.

Table S3. Search strings used in PubMed/MEDLINE, Scopus, and Web of Science Core Collection.

Database	Search string
PubMed/MEDLINE	("brain"[tiab] OR "cerebral cortex"[tiab] OR neurohistolog*[tiab] OR cytoarchitect*[tiab] OR Nissl[tiab] OR Golgi[tiab] OR immunohistochem*[tiab] OR stereolog*[tiab] OR morphometr*[tiab])<br><br>AND ("digital histology"[tiab] OR "whole-slide imaging"[tiab] OR "image analysis"[tiab] OR "machine learning"[tiab] OR "deep learning"[tiab] OR segmentation[tiab] OR "brain atlas"[tiab] OR "magnetic resonance imaging"[tiab] OR "diffusion tensor imaging"[tiab])<br><br>AND (dog*[tiab] OR canine[tiab] OR cat[tiab] OR cats[tiab] OR feline[tiab] OR horse*[tiab] OR equine[tiab] OR cattle[tiab] OR bovine[tiab] OR sheep[tiab] OR ovine[tiab] OR goat*[tiab] OR caprine[tiab] OR pig*[tiab] OR porcine[tiab] OR swine[tiab] OR cetacean*[tiab] OR dolphin*[tiab])<br><br>AND ("1990/01/01"[dp] : "2026/06/15"[dp])
Scopus	TITLE-ABS-KEY(brain OR "cerebral cortex" OR neurohistolog* OR cytoarchitect* OR Nissl OR Golgi OR immunohistochem* OR stereolog* OR morphometr*)<br><br>AND TITLE-ABS-KEY("digital histology" OR "whole-slide imaging" OR "image analysis" OR "machine learning" OR "deep learning" OR segmentation OR "brain atlas" OR "magnetic resonance imaging" OR "diffusion tensor imaging")<br><br>AND TITLE-ABS-KEY(dog* OR canine OR cat OR cats OR feline OR horse* OR equine OR cattle OR bovine OR sheep OR ovine OR goat* OR caprine OR pig* OR porcine OR swine OR cetacean* OR dolphin*)<br><br>AND PUBYEAR > 1989 AND PUBYEAR < 2027
Web of Science Core Collection	TS=(brain OR "cerebral cortex" OR neurohistolog* OR cytoarchitect* OR Nissl OR Golgi OR immunohistochem* OR stereolog* OR morphometr*)<br><br>AND TS=("digital histology" OR "whole-slide imaging" OR "image analysis" OR "machine learning" OR "deep learning" OR segmentation OR "brain atlas" OR "magnetic resonance imaging" OR "diffusion tensor imaging")<br><br>AND TS=(dog* OR canine OR cat OR cats OR feline OR horse* OR equine OR cattle OR bovine OR sheep OR ovine OR goat* OR caprine OR pig* OR porcine OR swine OR cetacean* OR dolphin*)<br><br>Timespan: 1990-01-01 to 2026-06-15 (set in the database interface)

Table S3: Search strings used for each bibliographic database (last search: 15 June 2026). Within each group of terms, synonyms were combined with the operator OR, and the three groups (brain and neurohistological/morphometric analysis; digital and computational methods and brain mapping; species of veterinary interest) were combined with the operator AND. [tiab], title/abstract (PubMed); TITLE-ABS-KEY, title, abstract, and keywords (Scopus); TS, topic, i.e., title, abstract, author keywords, and Keywords Plus (Web of Science). The asterisk (*) indicates truncation. Historical works published before 1990 were identified separately through Google Scholar, reference monographs, and the reference lists of the included studies (Section 2.4 of the main text).
